# Anterior sacral meningocele infected with Fusobacterium in a patient with recently diagnosed colorectal carcinoma – a case report

**DOI:** 10.1186/s12883-017-0992-1

**Published:** 2017-12-08

**Authors:** Anne K. Braczynski, Marc A. Brockmann, Torben Scholz, Jan-Philipp Bach, Jörg B. Schulz, Simone C. Tauber

**Affiliations:** 1 Department of Neurology, RWTH University Hospital, Pauwelsstr. 30, 52074 Aachen, Germany; 2 Department of Diagnostic and Interventional Neuroradiology, RWTH University Hospital, Aachen, Germany; 3 Department of Neurosurgery, RWTH University Hospital, Aachen, Germany

**Keywords:** Meningitis, Colorectal carcinoma, Anterior sacral meningocele, Occult spinal dysraphism

## Abstract

**Background:**

Anterior sacral meningoceles are rare, and usually occur with other malformations of the posterior lower spine. While these are more frequently reported in pediatric cohorts, we report a case in an elderly woman.

**Case presentation:**

We report on a 71 year-old woman with a recently diagnosed colorectal adenocarcinoma who presented with a severe bacterial meningitis. The cerebrospinal fluid cell count revealed a pleocytosis of 80,000 cells/μl and a severe disturbance of the blood-brain-barrier. *Fusobacterium nucleatum* was cultured as the causing pathogen. A lumbar MRI showed, in addition to contrast-enhancing meninges as sign of inflammation, a presacral mass. In the next step, the mass was diagnosed as an anterior sacral meningocele connected to the gut. An adequate antibiotic was used to treat the leptomeningitis. The connection between gut and meningocele was closed surgically and the patient recovered well and underwent further treatment of her colorectal adenocarcinoma.

**Conclusion:**

We report on a case of meningitis with an anterior sacral meningocele that was connected to the gut in a patient with a infiltrative colorectal adenocarcinoma. Anatomic variants have to be considered as rare causes of meningitis with typical intestinal germs.

## Background

Usually, a sacral meningocele develops from an incomplete closure of the neuroporus posterior. This so-called dysraphism is subcategorized according to its content: It is termed meningocele if it contains parts of the meninges; or myelocele, if spinal cord tissue is part of the herniated tissue and meningomyelocele, if it contains boths. These structures are usually dorsal deformations located at the posterior part of the lower back [1]. Nevertheless, anterior also termed pre-sacral myeloceles are described. Data on the incidence of anterior sacral myeloceles is not available. Clinically, patients present with bladder dysfunction and constipation. Most often, patients get already diagnosed during childhood.

## Case presentation

We report on a 71 year-old female patient who presented with fever, worsening of her general condition, neck stiffness, impaired vigilance and strong pain in the lower lumbar spine. Two weeks before, the patient had presented with weight loss and blood in her stool at a regional hospital. In a subsequent colonoscopy, a colorectal adenocarcinoma was diagnosed histologically. Staging consisting of a computed tomography (CT)-scan revealed infiltration into the uterus and also into *Os sacrum*. A CT-guided biopsy from the mass infiltrating Os sacrum also confirmed the carcinoma. The caregivers opted for a neo-adjuvant treatment plan including radio- and chemotherapy with capecitabine. Irradiation had been applied once. The neurologic examination at admission to our hospital revealed a somnolent patient with nuchal rigidity and bilateral positive Babinski sign, all of which had developed in less than 12 h.

**Fig. 1:**
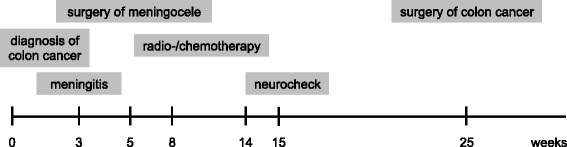
Case time line. This time line presents the most important clinical events in this case. The time scale is in weeks

Due to the clinical presentation, meningitis in the course of a spondylodiscitis was suspected. An antibiotic treatment with ceftriaxone and ampicilline was started, which lead to a rapid improvement of the patient’s consciousness. Extensive imaging including spinal magnetic resonance imaging (MRI), lumbar MRI, small pelvis MRI and CT scans of the lumbar spine and the brain were performed (results see Neuroradiological findings) and could rule out several differential diagnoses. There was no sign of spondylodiscitis. Analysis of the cerebrospinal fluid (CSF) revealed a pleocytosis of 80,000 cells/μl, dominated by neutrophilic granulocytes. Microbiologic work-up of CSF found the typical gastrointestinal germ *Fusobacterium nucleatum* as the causative agent of the bacterial infection, no other germs, e.g. *E. coli*, were detected. The antibiotic treatment was changed to ceftriaxone and vancomycine and continued for 14 days (see also Fig. 1).

### Neuroradiological findings

**Fig. 2:**
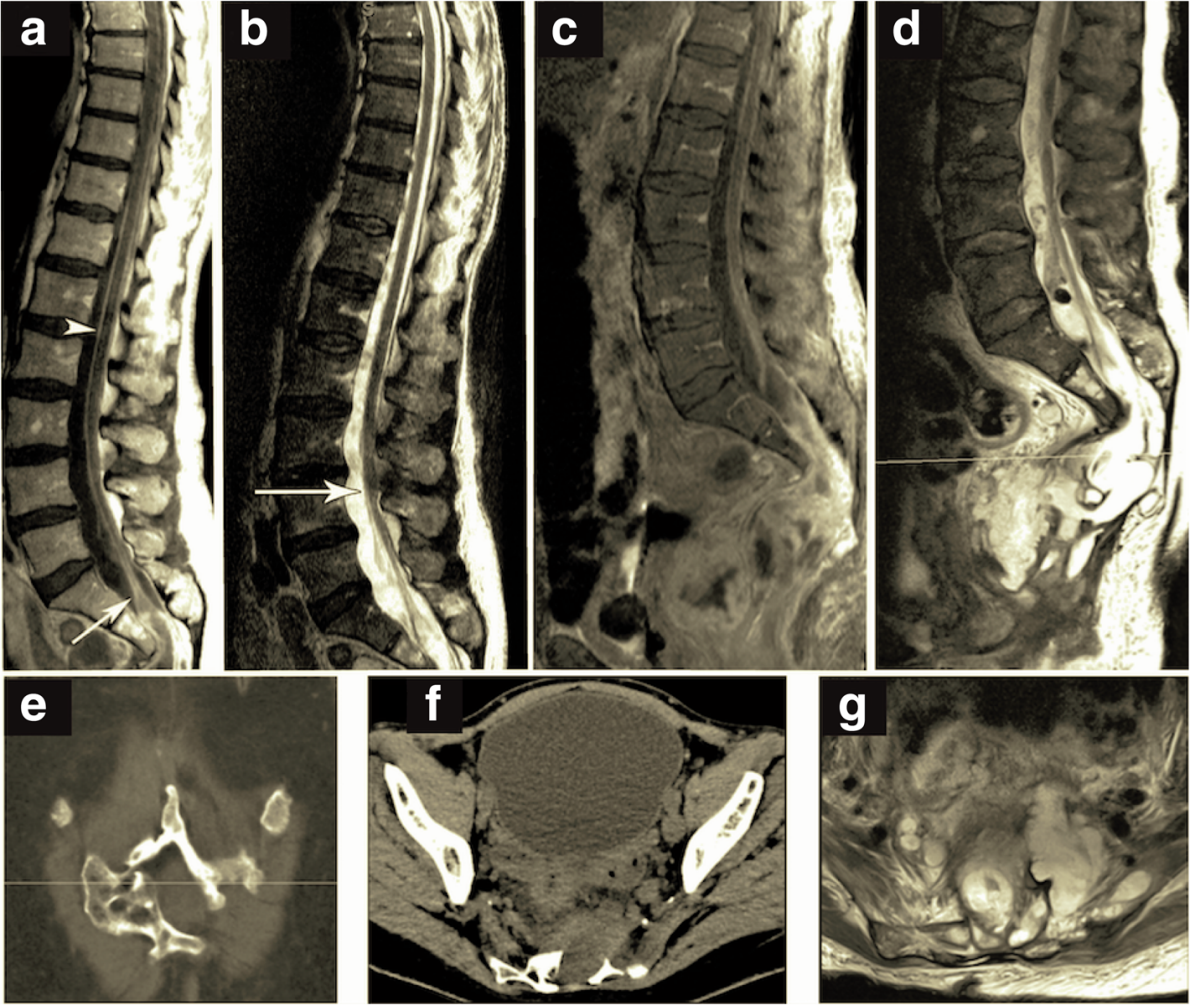
Neuroradiological Imaging. MRI imaging (**a** sagittal T1-SE post-contrast; **b** sagittal T2-TSE: sagittal fat-saturated T1-SE post-contrast; **d** sagittal T2-TSE, **e** coronally reconstructed CT; **f** axially reconstructed CT at the level marked by a line in **d**; **g** axial T2-TSE sequence at the level marked by a line in **c**). (**a**, **c**) Strong contrast enhancement of the meningeal structures due to the meningitis are shown in the post-contrast MRI series. The arrowhead in A exemplarily points at the strongly enhancing surface of the spinal cord. The arrow in A points at the contrast-enhancing filum terminale. (**b**) The level of the medullary cone is at the level of the vertebrates L3 and L4, which is unusually low, see arrow. (**c**) The distended rectum and a connection to the spinal canal are visible. The meningocele cannot be unequivocally delineated from the rectal carcinoma. (**d**, **e**) CT at the level marked by a line in **d**; (**f**) axial sequence at the level marked by a line in **c**) demonstrating a sacral menigocele with tethering of the spinal cord and bony dysraphism

Neuroradiological diagnostics including CT scans and MRI of the lumbar spinal column and the pelvis were performed. The CT-scan showed multiple spots of gas accumulation in the region of the lumbar vertebral disk between the vertebral bodies L4 and L5. MRI (Fig. 2) revealed, in addition to contrast-enhancing meninges illustrating the severe leptomeningits, a presacral mass filled with CSF isointens content. This mass could not be discriminated from necrotic cell debris. Initially, either necrotic tumor tissue of the adenocarcinoma or a malformation of the spinal dural sac seemed possible. Further anatomic abnormalities were an unusually low ending of conus of the spinal cord at L3/4 and a partial agenesis of the left sacral bone*.* A comprehensive work-up of the neuroradiological images revealed evidence of the presence of an anterior sacral meningocele. The carcinoma had infiltrated neighboring structures in the lower pelvis including bladder, and the sacral bone. Furthermore, a connection between the distended rectum and the spinal canal was visible. Through this pathway, bacterial invasion may have occurred and lead to a meningeal infection with typical gastrointestinal germ *Fusobacterium*. An interdisciplinary tumor board opted for surgical treatment to close the connection between gut and the anterior meningocele, which would prevent further pathogen invasion.

### Neurosurgical treatment

**Fig. 3:**
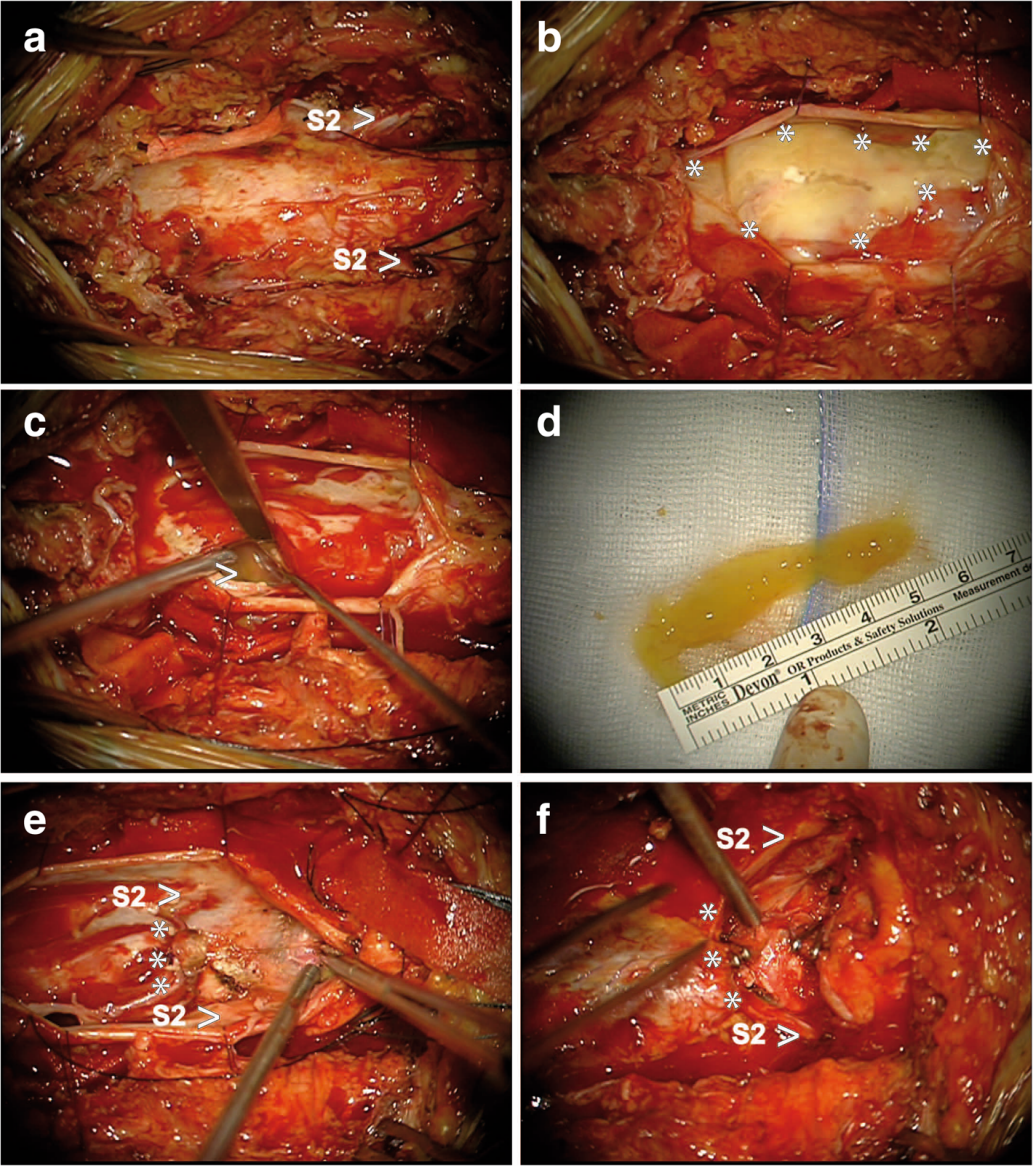
Neurosurgical intraoperative situs. Intraoperative findings. (**a**) Opening of the dural sac with spinal root of S2 (arrow). (**b**) Pus and necrotic cell debris (stars indicate the borders) were visible upon opening of the dural sac. (**c**) Cauda fibers adhered to each other due to the necrotic cell debris (arrow). (**d**) Extracted necrotic cell debris without any indication of tumor infiltration in the neuropathologic examination. (**e**, **f**) The intradural spinal root of S2 (arrow) and amputated cauda equina below S2 is visible (stars indicate amputation line)

To avoid recurrent meningitis, neurosurgical treatment was necessary. Since surgical therapy of the colorectal carcinoma after a neoadjuvant treatment would include creating terminal stomas of both bowel and urinary tracts, we opted for detachment of the local structures and closure of the thecal sac and amputation of the meningocele as far distal as possible. This was achieved distal of the S2-roots via a dorsal laminectomy from S1 to S3 (Fig. 3). We detached the cauda equina intradurally and sectioned and closed the thecal sac. Around and between adherent roots of the cauda equina postinflammatory debris was found. Moreover we could visualize the connection of the meningocele through the ventral defect of the *Os sacrum* into the small pelvis extra- and intradurally. The patient recovered well after surgery. Motor function was unchanged postoperatively. Bladder function could not be assessed due to an inserted urinary catheter. Histology of the surgical specimen showed meningeal cell layers invaded by leucocytes and macrophages.

### Oncologic treatment and follow up

Oncologic treatment consisting of radio- and chemotherapy resumed three weeks after the initial surgery. Radiotherapy of pelvis and *os sacrum* was applied in fractions of 1.8 Gy leading to a total of 54 Gy. In parallel, capecitabine was administered twice daily (825 mg/m^2^ body surface). At a clinical follow up 3 months later, the patient’s gait was normal, she denied abnormal functions of bladder or rectum. An abdominal CT-scan 24 weeks after initial admission showed necrotic consolidation of the tumor mass but a mild progression of lymph node size. In week 25, total mesorectal excision and ultralow anterior resection of the colon was performed. The patient had no children; there were no additional family history details. Siblings and parents were reported to be healthy. There were no other cutaneous or orthopedic stigmata pointing to a dystrophic condition. The patient had no foot deformities. The patient reported voiding problems, which had been present for many years. Unfortunately, the patient refused further genetic testing or urodynamic investigations (see also Fig. 1).

## Discussion and conclusion

We reported on a patient with known bladder dysfunction who developed severe leptomeningitis caused by *Fusobacterium nucleatum* shortly after the diagnosis of a colorectal adenocarcinoma. Neuroradiologic imaging lead to the diagnosis of a pre-existing anterior sacral meningocele. A presacral mass during staging via CT-scans at the time of the diagnosis of the colorectal cancer was first interpreted as extensive tumor mass. The anterior sacral meningocele and a connection into the colon was confirmed during surgery. The meningocele was accompanied by a tethered cord and partial agenesis of the left lower sacrum, thereby pointing to a complex developmental malformation.

**Fig. 4:**
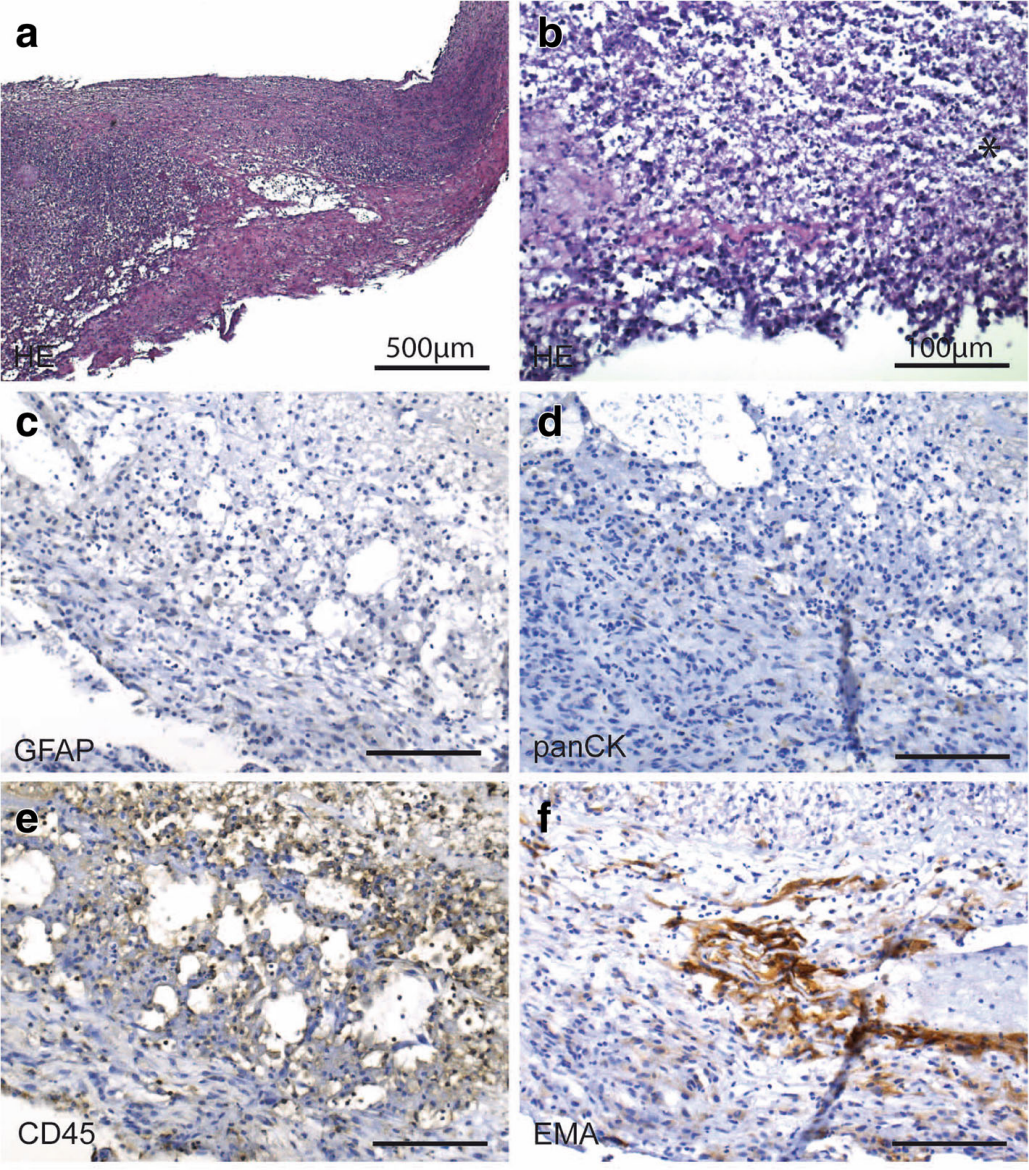
Neuropathologic findings. Histology of the tissue sample. (**a**) Overview of the tissue consisting of cell debris with sparse organisation (scale bar 500 μm, HE) and (**b**) detail (scale bar 100 μm). (**c**) Central nervous tissue (scale bar 100 μm, GFAP) as well as (**d**) infiltrative carcinoma cells (scale bar 100 μm, panCK) were absent. (**e**) The specimen were infiltrated by leukocytes and macrophages (scale bar 100 μm, CD45). (**f**) There were few EMA positive flat cells indicating presence of meningeal cell layer as a wall of the meningocele (scale bar 100 μm, EMA)

Finding a gut germ in the CSF was rather unusual. Different scenarios might explain its presence. First, a contamination of the biopsy needle during the CT-guided biopsy could have inoculated the germ into the CSF, therefore being an iatrogenic event. Still, a delay of three weeks between the biopsy and the onset of the meningitis leaves this option less probable. Second, in patients with the given malformations preformed colon-to-spinal fistulas are described. If such fistulas are preexisting, meningitis can occur and need a surgical closure, as a case in a one-month old girl infected with *E. coli* illustrates [2]. If a fistula might have been present in our patient, we postulate, that it might have gotten symptomatic at an earlier time point in live. Third, a hematogenic distribution of the germ through the venous plexus in the pelvis, that may contain anastomoses between colorectal veins and peridural veins, could have led to an infection. Lastly, we think that the colorectal adenocarcinoma formed an artificial connection between the meningocele and the colon, which was reported to exist during surgery. Unfortunately, the tumor invasion into the meningocele could not be proven histologically (Fig. 4), which might be explained by a sampling error. Both last options can explain the acute onset of the meningitis.

The patient admitted a bladder dysfunction being present a long time before the onset of the colorectal adenocarcinoma. Unfortunately, no further diagnostics had been performed earlier, and she denied urodynamic investigation after recovery. Patients with anterior sacral meningocele can suffer from pollakisuria because of bladder compression leading to a small capacity and can lead to obstetrical hindrance during pregnancy and delivery [2]. From a surgical and oncologic perspective, the presence of a postoperative bladder dysfunction is likely.

In patients with bladder dysfunction, sacral meningoceles were described in a pediatric cohort of 175 patients [3]. In 60% of the patients, they were associated with defects of the bone as present in our adult patient. Meningoceles can be part of the Currarino syndrome, which consists of a sacrococcygeal defect, a presacral mass (anterior meningocele and/or tumor, often teratoma) and an anorectal malformation. Currarino proposed this triad as a persistent neuroenteric malformation transmitted autosomal dominantly [4]. A mutation in the homeobox gene HLXB9 can be causative [5] and is found in about 50% of these patients [6]. A few reports describe case series with Currarino triad predominantly in pediatric cohorts [2, 7]. Nevertheless, while more than 80% of the cases are diagnosed within the first to third decade, Currarino syndrome may be diagnosed in older adults, especially if single features of the triad are missing due to incomplete penetrance [8]. The embryonic development of anterior sacral meningoceles also in the context of Currarino syndrome is not fully understood, it most probably results from a complex formation disorder. It is thought to develop in the course of the split notochord syndrome, a gastrulation defect, where persisting tissue parts are supposed to prevent the sufficient development of the vertebral bodies [9]. Given the triad of meningocele, sacral malformation and tethered cord, our patient possibly had suffered from Currarino syndrome. She reached a relatively old age at the time of diagnosis. Unfortunately, she refused further genetic testing to clarify the diagnosis. Genetic testing is advisable in such patients more importantly with respect to its autosomal-dominant inheritance. Evidence on the treatment of anterior meningoceles is very rare as well and a consensus on neurosurgical treatment is missing. In the case of a meningitis, surgery is well accepted [2]. Lower spinal imaging is advisable in patients which neurogenic bladder dysfunction, and a comprehensive presurgical neuroradiologic work-up is necessary in patients with this malformation.

In summary, anterior sacral meningoceles are underdiagnosed and account for differential diagnosis of anterior sacral masses. These anatomic variants have to be considered as rare causes of meningitis with typical intestinal germs. They need detailed neuroradiologic description, and lack established therapy strategies. We report on the distal sectioning of the thecal sac to close the anterior sacral meningocele for the prevention of recurrent meningitis.

## Abbreviations

CSF: Cerebrospinal fluid
CT: Computed tomography
MRI: Magnetic resonance imaging
